# Handwriting Tics in Tourette’s Syndrome: A Single Center Study

**DOI:** 10.3389/fpsyt.2016.00015

**Published:** 2016-02-17

**Authors:** Carlotta Zanaboni Dina, Alberto R. Bona, Edvin Zekaj, Domenico Servello, Mauro Porta

**Affiliations:** ^1^Tourette’s Syndrome and Movement Disorders Center, Galeazzi Hospital, Milan, Italy; ^2^Functional Neurosurgery Unit, Galeazzi Hospital, Milan, Italy

**Keywords:** handwriting, Tourette, tics, obsessions, compulsions

## Abstract

Tourette’s syndrome (TS) is a neurodevelopmental disorder typically defined by multiple motor tics and at least one sound tic, beginning in childhood or in adolescence. Handwriting is one of the most impaired school activities for TS patients because of the presence of tics that hamper learning processes. In this paper, we present a case of handwriting tics in a TS patient highlighting the main features.

## Tourette’s Syndrome

Tourette’s syndrome (TS) is a neurodevelopmental disorder typically defined by multiple motor tics and at least one sound tic (1), beginning in childhood or in adolescence.

More recently, TS has been acknowledged as a broad spectrum syndrome (2), including different comorbidities and coexisting symptoms. When beginning in early childhood TS mainly presents with attention deficit and hyperactivity disorder (ADHD) and tics, when beginning in adolescence instead tics and obsessive–compulsive behavior or disorder (OCB/OCD) are predominant. OCB/OCD trait is present in 60–80% of patients (3), and they are considered as thought tics (4). In many cases, motor and sound tics resolve spontaneously in adulthood, though OCB/OCD generally remains.

Tics often interfere with subject’s daily activities (5) affecting Quality of Life and causing Social Impairment, particularly in schooling and working. Handwriting is one of the most impaired school activities for TS patients because of the tics presence that hamper learning processes.

In our clinical experience, handwriting tics (HT) could severely affect and condition TS subjects, but they are not often pointed out in the Literature. For this reason, there are not precise data regarding the incidence of HT neither in TS patients nor in healthy population.

## Handwriting Tic Phenomenology and Differential Diagnoses

Patients suffering from TS may have different types of HT: (a) paligraphia, i.e., writing again and again the same letter, or word, or sentence (for instance the subject could write “today today today is a sunny day d d d d”), (b) outlining each letter multiple times (6–8), (c) pulling the pen back while writing (9, 10).

In some cases, HT can be considered simultaneously motor and obsessional because the subject complies with obsessions through tics, e.g., some patients have a lucky number and feel the urge to write the same sentence the lucky number of times.

From the differential diagnoses standpoint, HT have to be differentiated from other Written Expression Learning Disorders (1), such as dysgraphia, because of three different reasons. First, HT have a typical waxing and waning trend (bouts of tics) (9) whereas Written Expression Learning Disorders are constantly present. Second, unlike Written Expression Learning Disorders, HT respond to the same medications commonly used for tic management. Moreover, HT typically resolve after youth as many motor tics while Written Expression Learning Disorders may remain.

## Our Center Experience on Handwriting Tics

Given this rationale, we are conducting a study in our center to verify if patients with TS suffer from HT more than controls.

Handwriting tics study started out in Spring 2014. To date, the study has been conducted on 80 patients affected by TS, 58 males and 22 females. Patients’ age varies between 10 and 40 years (mean = 15 years old), and all patients have been followed by our multidisciplinary team of experts for at least 1 year. The age gap was chosen to include young people because of the higher prevalence of tics in youth. Patients have been identified by a single TS expert and by a single experienced neuropsychologist, who ruled out any Written Expression Learning Disorder case. Then authors have enrolled 35 non-patient primary Italian speaker controls living in Milan for comparison, 25 males and 10 females. Their age is 10–40 years, and the mean age is 16.

After being diagnosed, all patients have been treated with specific medications and/or habit reversal training (HRT) as cognitive-behavioral psychotherapy (11); medication intake and HRT response have been monitored during the entire study duration. We expected a 20% HT rate in TS subjects.

## Materials and Methods

This study has been carried out in accordance with the recommendations of “European Tourette Syndrome Guidelines,” (11) “Galeazzi Research and Clinical Hospital Committee” with written informed consent from all subjects. All subjects gave written informed consent in accordance with the Declaration of Helsinki.

At the time of first clinical assessment, patients have been evaluated in an off-medication state. An experienced neuropsychologist administered several tests that are: “Handwriting Assessment Test,” DCI (12), YGTSS (10), and YBOCS (13).

“Handwriting Assessment Test” was created “*ad hoc*” based on the clinical Italian experience, it consists of (a) a spontaneous writing subtest of 10 lines to be completed by the patient, and (b) a time-lapse subtest to analyze handwriting (graphics signs, pen handhold, page setting, and timing). Through this test, the clinician (the neuropsychologist) evaluates the presence/absence of HT and any improvement in writing in comparison with previous assessments.

DCI (12) measures TS percentage of diagnosis. We included patients with a minimum of 60% as score.

YGTSS (10) – tic severity and Social Impairment subscales – is the most common TS scale including the evaluation of HT. We include patients with a minimum of 30/50 as score of tic severity subscale and a minimum of 20/50 as score of Social Impairment subscale.

YBOCS (13) is the most worldwide used obsessive–compulsive disorder scale, including the assessment of compulsions such as repetitions of written letters, words, and sentences. We include all patients regardless the YBOCS score because HT is not always OCB/OCD related.

Clinical history interview has been collected as well, and the habit reversal training has been proposed in some patients. Ultimately, a single experienced neurologist decided for the entire medication plan.

Habit reversal training is an evidence-based (14) cognitive behavioral treatment that leads patient to be aware of the TS disease and it works on acceptance and self management of tics. The goal is to ameliorate Quality of Life. In the study, HRT consisted in 10 weekly sessions of 1 h with the patient and the caregiver; at the end of every session, homework was assigned to the patient in order to quicken the psychotherapy. Medication treatment followed Jankovich’ and Kurlan’s medication paradigm (15) and the European Guidelines (11), including symptomatic treatment such as alpha adrenergic agonists, antidopaminergic drugs, topiramate, and botulinum toxin.

After being enrolled in the study, each patient has been examined every 3 months by the neurologist and by the neuropsychologist for medication intake check-up and for the psychological assessment. During each visit, the aforementioned tests have been repeated. In the study, we have followed each patient for at least 12 months. Patients’ results have been compared with the 35 primary Italian speaker controls.

## Results

To date, we completed data collection on 66 enrolled patients, whereas the remaining 14 patients are still under investigation.

In these 66 TS patients (mean DCI score: 75%), we observed a 40% HT rate (32 subjects), 24 are males and 8 are females. In the control group, we have not found out any subject suffering from HT instead.

After 1-year treatment and follow-up, among those 32 affected patients, 18 patients (56%) resolved their HT, with a normalization (clinical judgment) at the “Handwriting Assessment Test.” As consequence, their YGTSS Social Impairment subscale improved on the average of 20 points out of 50 (from 30 to 10 points after the study). Furthermore, their YBOCS improved on the average of 7 out of 40 points (from 18 to 11 points after the study).

Remaining 14 patients (44%) ameliorate the intensity of their HT, with a “Handwriting assessment scale” improvement of 23% on the average (clinical judgment: from 83 to 60% after the study). In this subgroup, as consequence, YGTSS Social Impairment subtest improved on the average of 10 points out of 50 (from 40 to 30 points after the study). Moreover, YBOCS improved on the average of 4 out of 40 points (from 24 to 20 points after the study).

DCI test has not been repeated after the study because it is principally used as baseline diagnostic instrument.

## Conclusion

The goal of this study is to verify the presence of handwriting tics in patients suffering from TS and to assess the efficacy of medications and other aids (i.e., habit reversal training) in those treated. Milan Tourette’s Syndrome Center is still following all patients and collecting data.

As we mentioned, we expected a 20% HT rate in TS school-age subjects, but so far we found out a 40% HT rate instead. Given the absence of HT rate in controls, we observed a 40:0 HT ratio between patients and controls. HT have been predominant in males than in females.

Add-on treatments (medications and/or habit reversal training) have been helpful in 56% of patients suffering with HT; for this reason, we considered both clinical interventions effective in treating handwriting tics.

In our control group, we have not found out any subject suffering from HT, probably because of the very small number of subjects.

More studies are needed worldwide about handwriting tics and other specific tics.

## A Clinical Case

**Figure 1:**
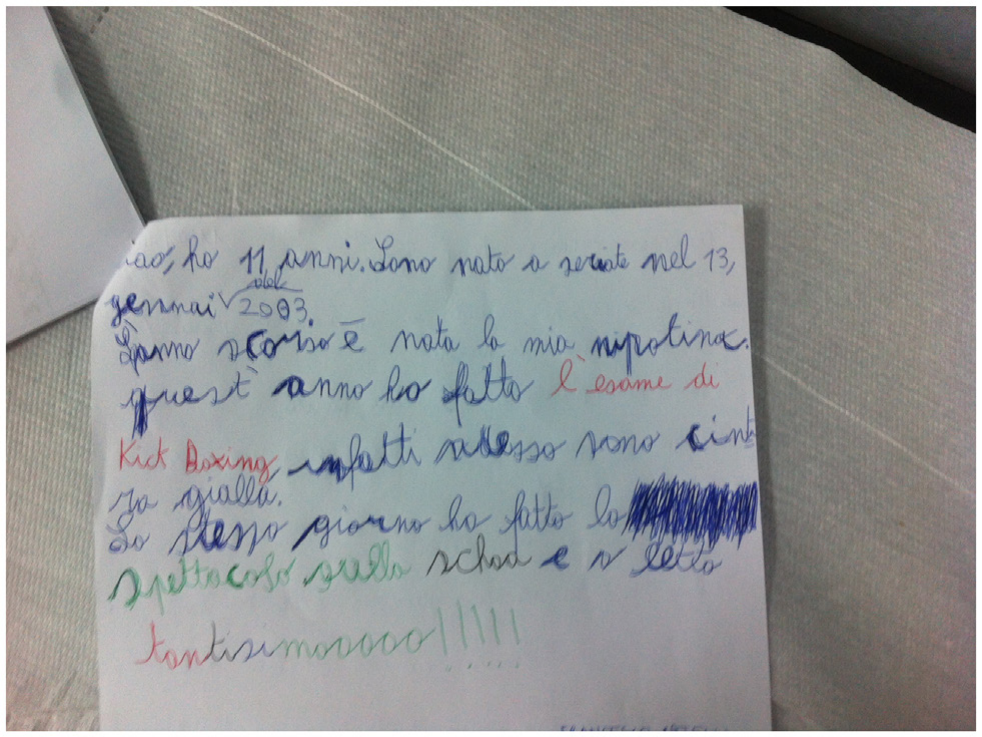
**A picture of handwriting tics of a Tourette’s syndrome subject (Marco) before treatments**.

In the following lines, we describe a TS clinical case of a child suffering from severely debilitating handwriting tics (Figure 1). Marco is an 11-year-old boy, attending the first year of a middle school in Milan. He was evaluated by our neurologist, who is an experienced TS specialist. At the first visit, he came to Milan Tourette’s Syndrome Center with neither medication nor psychological support. He was diagnosed of TS, and both medication intake (psychopharmacological treatment) and habit reversal training were implemented. HRT was implemented targeting the handwriting tic, and lasted 10 sessions, it was conducted between the therapist, the patient and Marco’s parents.

During the neuropsychological assessment, he displayed a severe handwriting tic pattern (to pull the pen back and to outline letters multiple times).

Marco’s Quality of Life was impaired, especially at school and with schoolmates. During lessons, Marco had to use a scholastic voice-software instead of writing.

**Figure 2:**
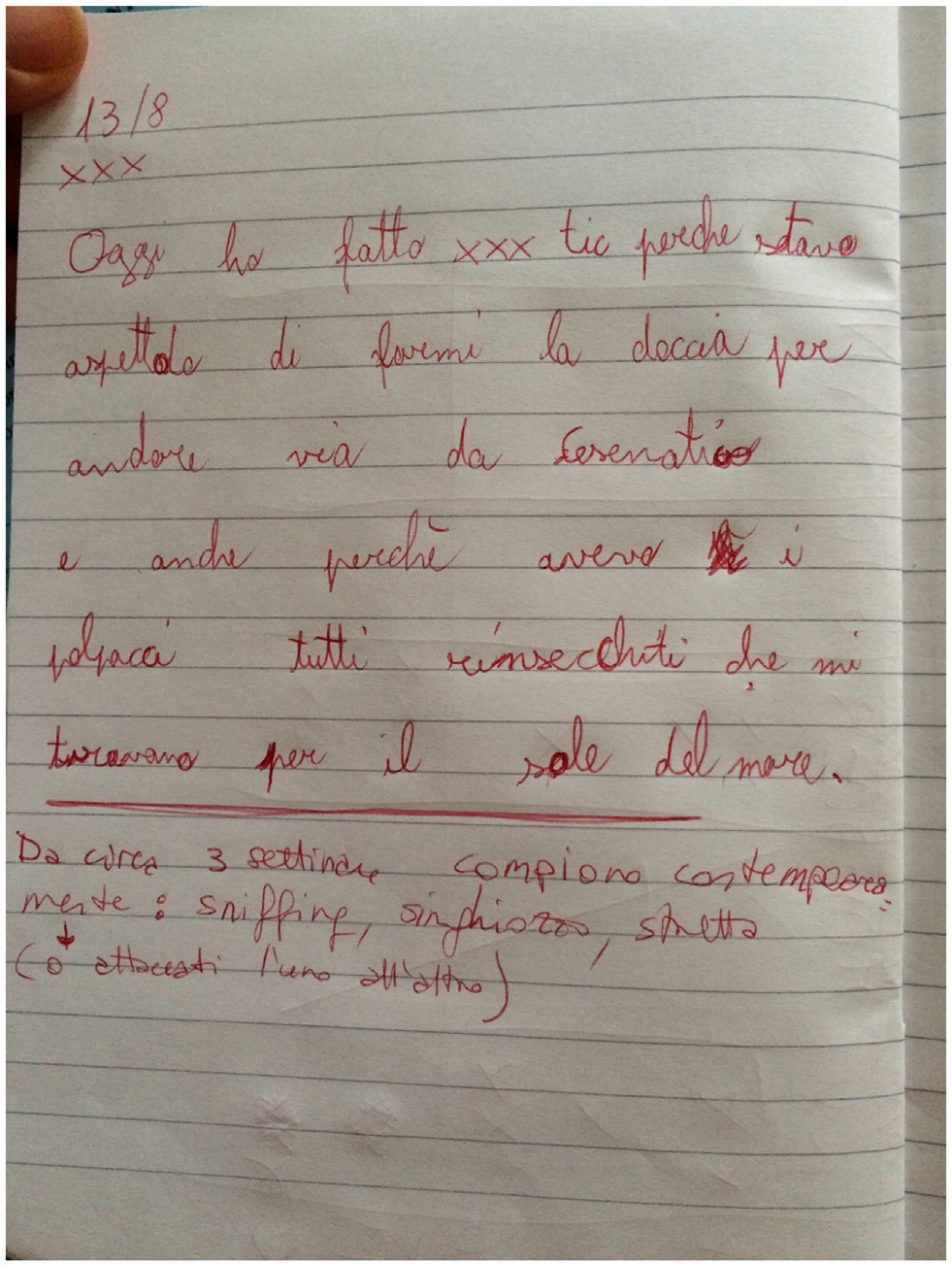
**A picture of handwriting tics of a Tourette’s syndrome subject (Marco) during treatments**.

After 2 months of treatments, Marco definitely improved his handwriting as reported in Figure 2.

After 3 months of treatments, Marco could finally come back to write correctly. Even socially, Marco benefits from the treatments because he suddenly felt “I am as my schoolmates.” Now Marco is well accepted in the class group and he recommends other TS subjects to be treated for their socially impairing tics.

## Author Contributions

CD visited patients as Tourette Syndrome expert neuropsychologist and wrote the manuscript, AB wrote the manuscript with CZ and ED, and DS elaborated data of the study, MP is the expert Tourette Syndrome neurologist who visited patients.

## Conflict of Interest Statement

The authors declare that the research was conducted in the absence of any commercial or financial relationships that could be construed as a potential conflict of interest.

## Abbreviations

HT, handwriting tics; HRT, habit reversal training.
